# Remember *Pfiesteria*?: Occupational Exposure Unlikely to Cause Cognitive Effects

**Published:** 2006-07

**Authors:** Tanya Tillett

Case reports have suggested that exposure to the dinoflagellate *Pfiesteria* may contribute to deficits in human learning and memory. Until now, however, there has been no clear, objective documentation of health effects associated with regular occupational exposure to this organism. The results of the first systematic, multiyear study of *Pfiesteria*’s human health effects now demonstrate that commercial fishermen (“watermen”) likely do not face significant health risks from routine occupational exposure to the organism **[*EHP* 114:1038–1043; Morris et al.]**.

*Pfiesteria* is a common inhabitant of estuarine waters in the U.S. mid-Atlantic region in the summer and fall. In 1997, watermen working along the Pocomoke River, an estuary off Chesapeake Bay, experienced a pattern of neuropsychological deficits in association with fish kills linked to *Pfiesteria* outbreaks. Researchers studying *Pfiesteria* in a lab environment had reported similar memory and learning deficits.

Using a cohort of 88 healthy watermen with regular occupational exposure to Chesapeake Bay waters and 19 controls with minimal contact to the waters (matched to the watermen by zip code, age, and educational level), a team of Maryland researchers collected data over four summers, from 1999 through 2002. They questioned the subjects biweekly about symptoms like those reported in the 1997 episode and about their exposure to the waters and to known chemical toxicants. Subjects were tested at the beginning and end of each summer season on sensory and motor functions, attention and concentration, memory, visual functions, and verbal functions. In addition, the research team analyzed more than 3,500 water samples taken from Chesapeake Bay to monitor the presence of *Pfiesteria* and other harmful species.

*P. piscicida* was found in water samples drawn from a number of locations in all four years of the study, and *P. shumwayae* (recently renamed *Pseudopfiesteria shumwayae*) was found in the last two years. However, the investigators found no decline in neurological function among the watermen in any year of the study.

The scientists note that unique, isolated instances of *Pfiesteria* outbreaks or unusually toxic strains of the dinoflagellate may have been associated with the marked, reversible health effects documented in the past. They point out that the present study is congruent with similar studies in North Carolina and Virginia in providing reassurance that in the absence of these conditions, watermen do not appear to face significant health risks from routine occupational exposure to estuarine waters that contain *Pfiesteria*.

## Figures and Tables

**Figure:**
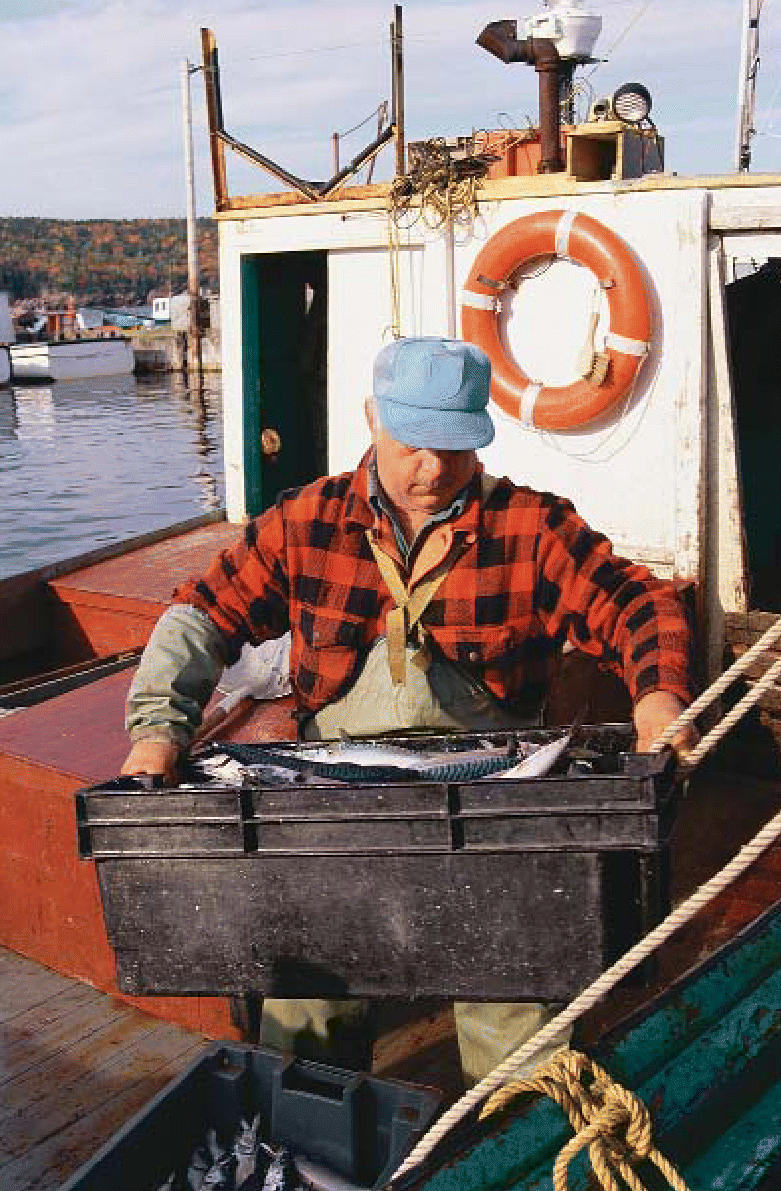
Safe from *Pfiesteria* New data suggest commercial fishermen need not fear routine exposure to the organism.

